# The complete mitochondrial genome of *Yuukianura szeptyckii* Deharveng & Weiner 1984 (Collembola: Neanuridae)

**DOI:** 10.1080/23802359.2021.1888330

**Published:** 2021-03-16

**Authors:** June Wee, Yun-Sik Lee, Taekjun Lee, Philjae Kim, Jino Son, Yongeun Kim, Kijong Cho

**Affiliations:** a Division of Environmental Science and Ecological Engineering, Korea University, Seoul, Republic of Korea; b O-Jeong Resilience Institute, Korea University, Seoul, Republic of Korea; c Marine Biological Resource Institute, Sahmyook University, Seoul, Korea; d Division of Ecological Conservation, Bureau of Ecological Research, National Institute of Ecology, Choongnam, Korea; e Biological and Genetics Resources Assessment Division, National Institute of Biological Resources, Incheon, Republic of Korea

**Keywords:** Springtail, gene order, phylogeny, mitogenome

## Abstract

The complete mitochondrial genome of *Yuukianura szeptyckii* Deharveng & Weiner 1984 was sequenced, assembled, and annotated. The mitochondrial genome of *Y. szeptyckii* has a length of 15,771 bp and contains 13 protein-coding genes (PCGs), 22 transfer (tRNA) genes, and 2 ribosomal RNA (rRNA) genes. *Y. szeptyckii* was closely clustered with the following species of Neanuridae: *Bilobella aurantiaca* and *Friesea grisea.*

Collembola are the most abundant organisms in the soil ecosystem and play an important role in nutrient cycling (Rusek 1998; Filser 2002). The family Neanuridae is a diverse family of Collembola that are characterized by the absence of the mandibular plate (Hopkin 1997). Approximately 1,600 species have been reported worldwide (Bellinger 1996–2020), but only a few complete mitochondrial genome records belong to the Neanuridea group (NCBI, accessed 2020 Oct 29; Dong et al, 2020). In the present study, sequencing, assembly, and annotation of the mitochondrial genome of *Yuukianura szeptyckii* were performed and their molecular characteristics were described.

Specimens of *Y. szeptyckii* were first collected from near a stream of Ansan City, Gyunggi province, Korea (37.288°N, 126.822°E) on 15 June 2006. Since then, *Y. szeptyckii* has been cultured in the laboratory for approximately 14 years. The DNA of the sample used in the present study was deposited at the Korea University (http://insect.korea.ac.kr, ssamppong@korea.ac.kr, specimen accession number: KUEMCOL002). Mitochondrial DNA was extracted using the Qproteome® Mitochondria Isolation kit (Qiagen, Hilden, Germany) according to the manufacturer’s instructions and isolated using a QIAamp DNA mini kit (Qiagen). Mitochondrial DNA was amplified using the REPLI-g Mitochondrial DNA Kit (Qiagen). Next-generation sequencing (NGS) analysis was performed using genome analysis units at the National Instrumentation Center for Environmental Management of Seoul National University in Korea. A genomic library was constructed from the genomic DNA using a Kapa Hyper Prep Kit (Kapa Biosystems, Woburn, MA, USA) using paired-end reading, which was followed by next-generation sequencing (NGS) on the Illumina Hi-Seq 2500 platform (San Diego, CA, USA). The complete mitogenome was annotated using the Geneious ver. 11.1.5 (Biomatters Ltd, Auckland, New Zealand). Transfer RNA (tRNA) genes were identified using tRNAscan-SE online (Lowe and Chan 2016), with the following search mode: the sequence source was ‘other mitochondrial’, and the genetic code for tRNA isotype prediction was ‘Invertebrate Mito’. Mitogenome sequences were aligned using MAFFT (Katoh and Standley 2013) and the mitogenome dataset was analyzed using maximum likelihood (ML) with RAxML 8.2 (Stamatakis 2014). The best-fit substitution was estimated using jModelTest 2.1.1 (Guindon and Gascuel 2003; Darriba et al. 2012) for the nucleotide dataset of 13 protein-coding genes (PCGs). For ML analyses, bootstrap resampling was performed using the rapid option with 1000 iterations.

The mitogenome of *Y. szeptyckii* is 15,771 bp long and contains 13 protein-coding genes (PCGs), 22 transfer (tRNA) genes, and 2 ribosomal RNA (rRNA) genes. The overall nucleotide composition was 34.0% A, 15.6% C, 10.1% G, and 40.3% T, indicating an obvious A + T bias (74.3%). For the 13 PCGs, three start codons are found: ATG (ATP6, COX3, ND4), ATT (ND2) and other nine PCGs has ATA. The ‘TAG’ stop codon reveal COX3, CYTB, ND5 and ND6, and other nine PCGs has ‘TAA’.

The gene arrangement of *Y. szeptyckii* was the same as that of the two other species of the Neanuradea family from GenBank (*Bilobella aurantiaca* (NC_011195) and *Friesea grisea* (NC_010535)); the gene arrangement of Collembola mitogenomes was relatively conserved compared to other taxa, including *Y. szeptyckii*. In the phylogenetic tree of ML, *Y. szeptyckii* clustered a sister group with closely related species that belonged to the family Neanuridae (*B. aurantiaca* and *F. grisea* (Figure 1)) and to a monophyletic clade of order Poduromorpha (Figure 1).

**Figure 1.**
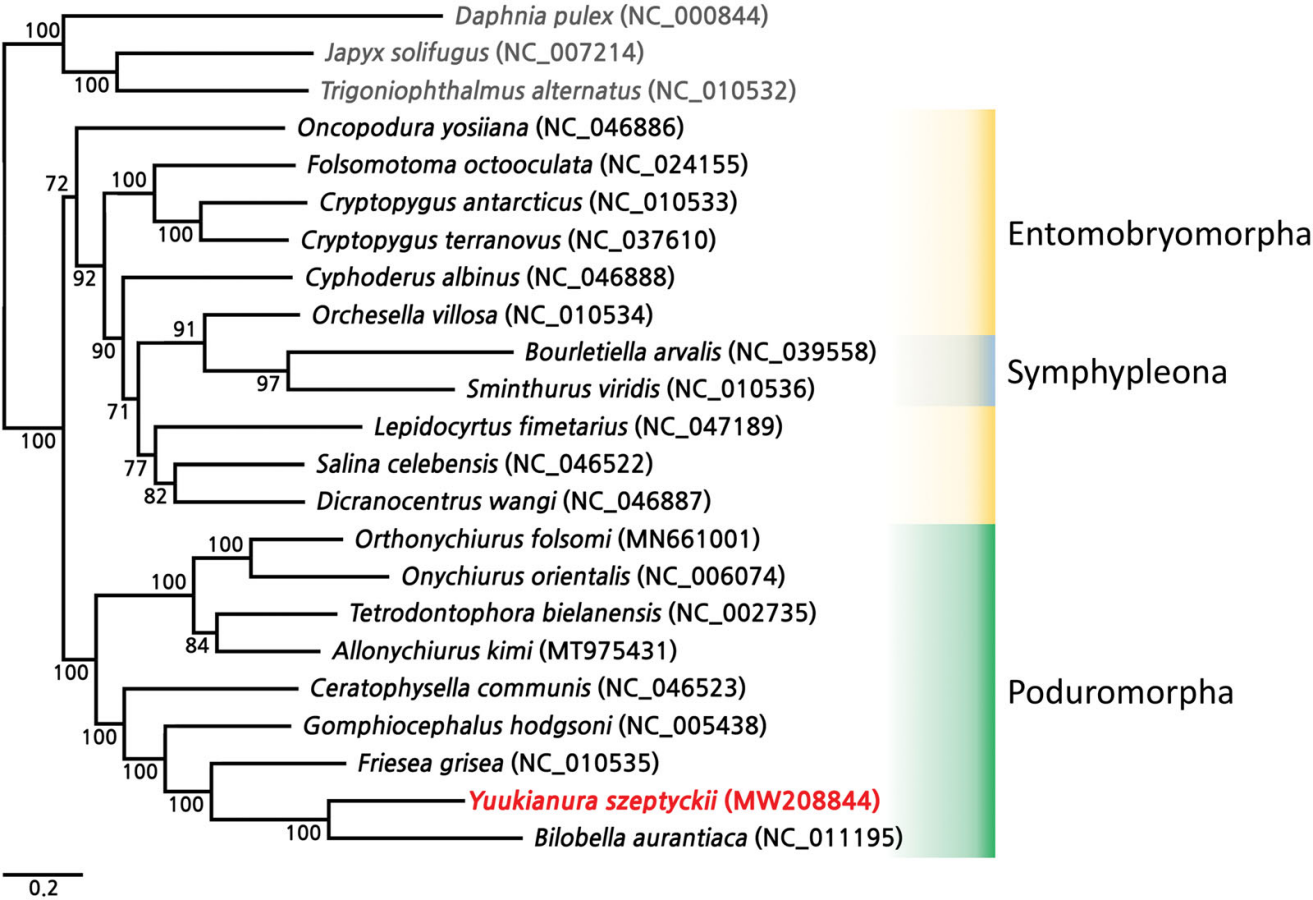
Phylogenetic tree of *Yuukianura szeptyckii* and 19 springtails from GenBank developed using the maximum likelihood method based on the nucleotide sequences of 13 protein-coding genes. The bootstrap support values are indicated on each node.

## Data Availability

The genome sequence data that support the findings of this study are openly available in GenBank of NCBI at (https://www.ncbi.nlm.nih.gov/) under the accession no. MW208844. The associated BioProject, SRA, and Bio-Sample numbers are PRJNA694843, SRR13529482, and SAMN17575579, respectively.
